# Clinical Reliability of AI-Based Cephalometric Analysis Using WebCeph: A Comparative Agreement Study

**DOI:** 10.3390/jcm15114155

**Published:** 2026-05-28

**Authors:** Ali Azari-Mehr, Angela Bisbal-Puchades, Laura Marqués-Martínez, Maria Carmona-Santamaria, Esther García-Miralles, Juan Ignacio Aura-Tormos, Clara Guinot-Barona

**Affiliations:** 1 Dentistry Department, Medicine and Health Science Faculty, Catholic University of Valencia, 46001 Valencia, Spain; alia@mail.ucv.es (A.A.-M.); anbispu@mail.ucv.es (A.B.-P.); maria.carmona@ucv.es (M.C.-S.); clara.guinot@ucv.es (C.G.-B.); 2 Stomatology Department, University of Valencia, 46010 Valencia, Spainm.esther.garcia@uv.es (E.G.-M.)

**Keywords:** artificial intelligence, cephalometry, orthodontics, diagnostic imaging, reproducibility of results

## Abstract

**Background/Objectives**: Artificial intelligence has accelerated cephalometric analysis by enabling rapid and standardized measurements. However, whether these automated outputs can be considered clinically interchangeable with expert manual tracing remains unresolved, particularly for routinely used analyses such as Steiner. **Methods**: A comparative experimental study was conducted on 100 lateral cephalometric radiographs analysed using two parallel approaches: expert manual tracing and fully automated analysis with the WebCeph platform. Seven Steiner variables (SNA, SNB, ANB, 1–NA, 1–NB, interincisal angle, and FMA) were evaluated. Paired *t*-tests were used to assess differences between methods, while agreement was evaluated using intraclass correlation coefficients and Bland–Altman analysis. Particularly low agreement was observed for clinically relevant parameters such as ANB and FMA. **Results**: Six of the seven variables showed statistically significant differences between methods. Automated measurements systematically tended to overestimate both skeletal and dental parameters. Agreement was inconsistent and frequently poor, with ICC values ranging from 0.01 to 0.60 for clinically relevant variables such as ANB and FMA. Importantly, small or non-significant mean differences did not translate into acceptable agreement. Bland–Altman analysis confirmed the presence of systematic bias and wide limits of agreement, especially for dental measurements. **Conclusions**: Despite its speed and automation, WebCeph does not achieve clinically acceptable agreement with expert manual tracing across several key cephalometric variables. The observed discrepancies—particularly in parameters critical for sagittal and vertical diagnosis—may compromise clinical interpretation and treatment planning. These findings support the use of AI-based cephalometric analysis as an adjunctive tool rather than a substitute for clinician-guided evaluation.

## 1. Introduction

Cephalometric analysis continues to play a central role in orthodontic diagnosis and treatment planning, offering a structured assessment of craniofacial morphology, sagittal and vertical skeletal relationships, dentoalveolar inclinations, and growth patterns derived from lateral cephalometric radiographs [1,2,3]. Although three-dimensional imaging has expanded diagnostic possibilities, conventional two-dimensional cephalometry remains widely used in daily practice. Its accessibility, standardized protocols, and the availability of long-established normative data continue to support its clinical relevance. In this context, cephalometric measurements inform key decisions, including extraction strategies, timing of orthopaedic interventions, selection between camouflage and surgical approaches, and evaluation of treatment outcomes [4,5].

The reliability of any cephalometric analysis ultimately depends on accurate identification of anatomical landmarks. Even minor deviations in landmark localisation may translate into clinically meaningful differences in angular or linear measurements, particularly in borderline cases where diagnostic thresholds are narrow. Evidence consistently indicates that both inter- and intra-operator variability contribute to measurement error in manual tracing, especially for landmarks associated with incisor apices, mandibular planes, and cranial base structures. Manual tracing is therefore commonly used as the clinical reference method, although it remains susceptible to methodological variability [6,7].

Among the various analytical approaches, Steiner analysis is still one of the most commonly applied systems in both clinical practice and orthodontic education. Its focus on sagittal skeletal relationships (SNA, SNB, ANB), incisor position relative to basal bone (1–NA, 1–NB), interincisal angle, and vertical growth pattern (Frankfort-mandibular angle) makes it particularly influential in treatment planning. Small variations in parameters such as ANB or incisor measurements may alter diagnostic categorization or affect decisions regarding extractions. For this reason, any automated system intended to reproduce Steiner analysis should be evaluated not only in terms of statistical agreement but also in terms of its clinical reliability at the individual patient level [8,9].

Artificial intelligence has recently been incorporated into orthodontic workflows through automated cephalometric platforms capable of detecting landmarks and generating measurements without direct operator input. This development reflects a broader transformation across dental disciplines, where AI applications have been increasingly explored to improve diagnostic efficiency and standardization. Most of these systems rely on deep learning architectures designed to reduce operator-dependent variability while improving consistency and facilitating integration into digital workflows [3,5].

A growing body of evidence indicates that deep learning–based approaches can achieve clinically acceptable levels of accuracy in cephalometric landmark detection under controlled conditions. However, reported performance varies depending on model architecture, training datasets, imaging conditions, and the specific anatomical landmarks analysed, with greater variability consistently observed in dental and complex anatomical regions [10,11,12,13,14,15,16].

However, the available evidence is not entirely consistent. Some studies report discrepancies between automated and manual measurements that exceed thresholds generally considered clinically acceptable, especially in angular variables relevant to sagittal and vertical assessment [17]. In addition, factors such as patient positioning, image quality, and anatomical variability appear to influence landmark identification, which may limit the robustness of automated outputs in routine clinical settings [18]. A further limitation of the current literature is the emphasis on mean differences or statistical significance, often without a detailed assessment of agreement. Similar average values do not necessarily imply that two methods are interchangeable, particularly when limits of agreement are wide or systematic bias is present [19,20,21,22,23,24,25].

WebCeph (https://webceph.com/es/) is a widely used web-based platform that performs fully automated cephalometric analysis through AI-driven landmark detection. Its ease of use and integration into digital workflows have contributed to its growing adoption in orthodontic practice. Nevertheless, the evidence regarding its performance when specifically applied to Steiner analysis remains limited. Existing studies on WebCeph and comparable systems suggest acceptable levels of accuracy in controlled environments but also point to variability across specific measurements and the continued need for clinician oversight [22,23,24]. In addition, relatively few studies have combined the analysis of mean differences, intraclass correlation coefficients, and Bland–Altman agreement to assess whether automated outputs can be considered clinically interchangeable with expert manual tracing [25,26,27].

Given the increasing digitalization of orthodontic diagnostics, a careful and methodologically robust evaluation of automated cephalometric systems is required before their independent clinical use can be justified. The aim of the present study was therefore to compare cephalometric measurements obtained through expert manual tracing with those generated automatically by the WebCeph platform using Steiner analysis, with particular attention to agreement, systematic bias, and clinical interchangeability at the individual level.

## 2. Materials and Methods

### 2.1. Study Design

This study was designed as a method comparison study with agreement analysis, conducted in accordance with the STROBE guidelines for observational studies [28]. The objective was to evaluate both statistical agreement and clinical interchangeability between expert manual cephalometric tracing and fully automated cephalometric analysis performed using an artificial intelligence–based platform (WebCeph).

**Figure 1:**
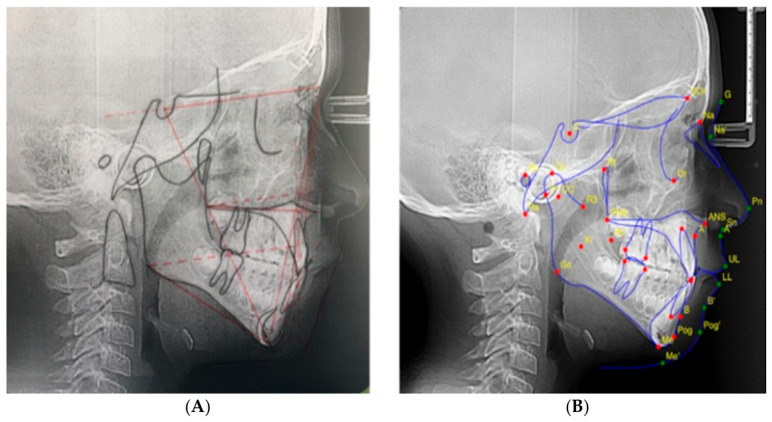
Representative comparison between expert manual tracing and fully automated cephalometric analysis: (**A**) Manual tracing performed by an experienced orthodontist, illustrating landmark identification and construction of Steiner measurements. (**B**) Automated analysis generated by the WebCeph platform, showing AI-based landmark detection and the corresponding cephalometric framework.

A within-subject design was implemented, whereby each lateral cephalometric radiograph was analysed using both methods under standardized conditions, allowing direct paired comparison of measurements. Representative examples of manual and automated cephalometric tracings are shown in Figure 1.

### 2.2. Study Sample

The study sample consisted of 100 lateral cephalometric radiographs obtained from patients treated at university dental clinics. Radiographs were randomly selected from the institutional database and anonymized prior to analysis.

Eligible radiographs corresponded to patients aged between 11 and 25 years, with diagnostic-quality lateral cephalograms presenting adequate contrast and resolution for reliable landmark identification and no history of previous orthodontic treatment. Radiographs were excluded if patients presented severe craniofacial anomalies or syndromes, had undergone orthognathic surgery, or if the images showed positioning errors, motion artefacts, or insufficient image quality that could compromise cephalometric analysis.

Particular attention was paid to radiographic standardization and image quality, given their known influence on AI-based landmark detection performance. Radiographs presenting metallic artefacts, motion blur, inadequate head positioning, significant asymmetry caused by rotation, low contrast, poor sharpness, or incomplete visualization of relevant anatomical structures were excluded from the study. In addition, radiographs with severe dentofacial deformities or anatomical conditions potentially compromising reliable landmark identification were not included.

All radiographs were acquired following standardized radiographic protocols in accordance with institutional clinical guidelines. All lateral cephalometric radiographs were acquired using the same digital cephalometric unit (Sirona Dental Systems GmbH, Bensheim, Germany) under standardized institutional acquisition protocols, with exposure parameters maintained within conventional orthodontic imaging ranges (approximately 70–80 kVp and 8–12 mA). This standardization aimed to minimize variability in image quality and reduce potential confounding effects on AI-based landmark detection.

The sample included patients with different skeletal and dentofacial characteristics representative of routine orthodontic diagnostic records obtained in university clinical practice.

### 2.3. Sample Size Estimation

An a priori sample size estimation was performed based on angular cephalometric measurements. A minimum clinically relevant difference of 2° was assumed, with an estimated standard deviation of 4°, a significance level (α) of 0.05, and a statistical power of 80%. Under these conditions, the minimum required sample size was calculated to be 64 radiographs.

To ensure adequate statistical robustness across all evaluated variables, a final sample of 100 lateral cephalograms was included.

### 2.4. Manual Cephalometric Tracing Protocol

Manual cephalometric tracing was performed by an orthodontist with 15 years of clinical experience in cephalometric analysis. Prior to data collection, a calibration process was conducted to standardize landmark identification criteria.

All tracings were performed under controlled viewing conditions, including standardized ambient lighting, calibrated high-resolution monitors, and fixed magnification settings to minimize variability. Landmark identification followed the original definitions proposed by Steiner, supported by a reference cephalometric atlas to ensure consistency in anatomical interpretation.

Angular and linear measurements were derived directly from the identified landmarks in accordance with Steiner analysis specifications. All manual tracings were initially performed by the primary examiner. Subsequently, the tracings were independently reviewed by a second experienced orthodontist blinded to the automated results. In cases where clinically relevant discrepancies in landmark identification or measurement interpretation were detected, a consensus review was performed jointly by both examiners according to predefined anatomical criteria based on Steiner landmark definitions and a standardized cephalometric reference atlas. Final manual values used for analysis corresponded to the consensus measurements established after this review process.

### 2.5. Reliability of Manual Tracing

Intra-observer and inter-observer reliability of the manual cephalometric analysis were assessed using a randomly selected subgroup of 20 lateral cephalograms (20% of the total sample), randomly selected from the overall dataset for repeated reliability assessment purposes.

For intra-observer reliability, the primary examiner repeated the tracing and measurements after a 3-week interval under identical conditions. For inter-observer reliability, a second independent orthodontist with 21 years of clinical experience performed the same analysis, blinded to the results of the first examiner.

Manual tracing reliability was reinforced through examiner calibration, repeated verification procedures, and independent review by a second experienced orthodontist. Standardized tracing conditions and consensus verification in cases of disagreement were implemented to minimize operator-dependent variability and strengthen the reproducibility of the manual reference measurements used for comparison with WebCeph.

### 2.6. Automated Cephalometric Analysis

Automated cephalometric analysis was performed using the WebCeph online platform (WebCeph, Republic of Korea), which applies convolutional neural network–based algorithms for fully automated landmark detection. All automated analyses were performed using the WebCeph online platform (AssembleCircle Corp., Seoul, Republic of Korea), accessed in December 2025 through the web-based interface available at the time of analysis. Because WebCeph operates as a continuously updated cloud-based platform, a specific software version number was not publicly available through the user interface or documentation. To ensure methodological consistency, all radiographs were analysed using the same platform release, default settings, and identical operating conditions throughout the study period, without software updates or user modifications during data collection.

Each lateral cephalometric radiograph was uploaded in its original digital format, and the software automatically identified cephalometric landmarks and generated corresponding angular and linear measurements according to Steiner analysis. No manual adjustment, correction, or operator intervention was performed at any stage of the automated analysis.

The WebCeph platform automatically performed landmark detection and scale calibration using the embedded radiographic reference system provided in the original digital cephalograms. No additional manual calibration or operator adjustment was performed during image processing or measurement generation.

### 2.7. Cephalometric Variables

Seven cephalometric variables were selected for comparison between manual and automated methods due to their established diagnostic relevance and routine clinical use. These included sagittal skeletal parameters (SNA, SNB, and ANB), dental measurements (1–NA and 1–NB, expressed in millimetres), the interincisal angle (1/1), and the Frankfort–mandibular plane angle (FMA), reflecting the vertical growth pattern.

These variables were chosen because of their direct influence on orthodontic diagnosis and treatment planning, particularly in skeletal classification, incisor positioning, and extraction decision-making.

### 2.8. Statistical Analysis

Statistical analysis was performed using IBM SPSS Statistics software (version 26.0; IBM Corp., Armonk, NY, USA). Mean values and standard deviations were calculated for each cephalometric variable for both manual and automated analyses. Data normality was assessed prior to inferential testing.

Differences between methods were evaluated using paired Student’s *t*-tests, with the level of statistical significance set at *p* < 0.05. Agreement between manual and automated measurements was assessed using intraclass correlation coefficients (ICCs) for absolute agreement.

In addition, agreement and potential systematic bias were evaluated using the Bland–Altman method by calculating mean differences (automated − manual) and 95% limits of agreement (mean ± 1.96 × standard deviation of the differences).

Beyond statistical significance, particular emphasis was placed on agreement analysis and the clinical relevance of observed differences, recognizing that statistical similarity does not necessarily imply clinical interchangeability.

### 2.9. Ethical Considerations

This study was conducted in accordance with the principles of the Declaration of Helsinki. All radiographs were originally obtained for routine diagnostic and treatment planning purposes in orthodontic care and were retrospectively collected from the institutional database.

Prior to analysis, all radiographs were fully anonymized to ensure patient confidentiality and data protection. According to Spanish national regulations on biomedical research (Law 14/2007, BOE-A-2007-12945) and institutional policies, retrospective studies using anonymized diagnostic data obtained for clinical purposes do not require formal ethics committee approval.

No additional radiographic exposure or intervention was performed for research purposes.

## 3. Results

### 3.1. Sample Characteristics and Analytical Overview

A total of 100 lateral cephalometric radiographs fulfilling the predefined inclusion criteria were included in the analysis. Each radiograph underwent two parallel evaluation procedures: expert manual cephalometric tracing and fully automated analysis using the WebCeph platform.

Seven Steiner cephalometric variables were assessed for each image, allowing direct within-subject comparison between manual and automated measurements.

### 3.2. Comparison of Mean Values Between Manual and Automated Analyses

Descriptive statistics and between-method comparisons are summarized in Table 1.

Across most variables, automated measurements were higher than those obtained through manual tracing. Statistically significant differences were observed in six of the seven evaluated parameters.

For skeletal measurements, both SNA and SNB showed higher values in automated analysis, with mean differences exceeding 3°. In contrast, ANB showed similar mean values between methods and did not reach statistical significance; however, agreement at the individual level remained poor.

Differences were more pronounced in dental measurements. Both 1–NA and 1–NB showed higher values in automated analysis, with mean differences of +1.65 mm and +2.29 mm, respectively. The interincisal angle also showed higher values in automated measurements.

For FMA, the mean difference was smaller (+0.94°) but remained statistically significant.

### 3.3. Agreement Analysis and Measurement Variability

Agreement between manual and automated measurements was assessed using intraclass correlation coefficients (ICCs), and Bland–Altman agreement parameters are presented in Table 2.

Moderate agreement was observed for SNB (ICC = 0.60), SNA (ICC = 0.55), and the interincisal angle (ICC = 0.52). Dental linear measurements showed lower agreement, with ICC values of 0.40 for 1–NA and 0.32 for 1–NB.

ANB showed very low agreement (ICC = 0.10) despite similar mean values between methods. FMA showed minimal agreement (ICC = 0.01).

**Figure 2:**
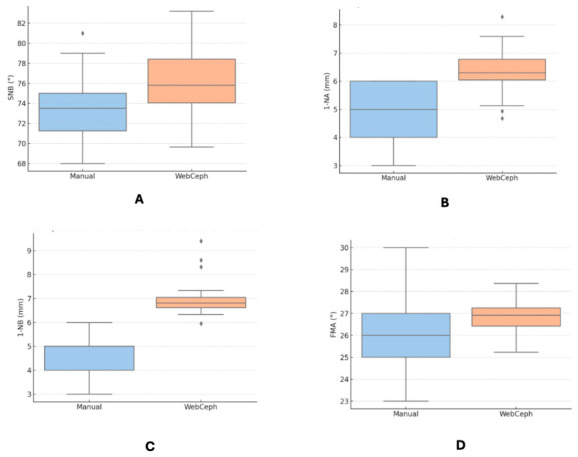
Distribution of cephalometric measurements obtained through manual tracing and automated analysis (WebCeph). Boxplots represent median values, interquartile ranges, and outliers for each variable: (**A**) SNB (°); (**B**) 1–NA (mm); (**C**) 1–NB (mm); (**D**) FMA (°). Across all variables, automated measurements tend to show higher central values compared with manual tracing.

Boxplots illustrating the distribution of selected variables are presented in Figure 2.

### 3.4. Bland–Altman Analysis and Systematic Bias

**Figure 3:**
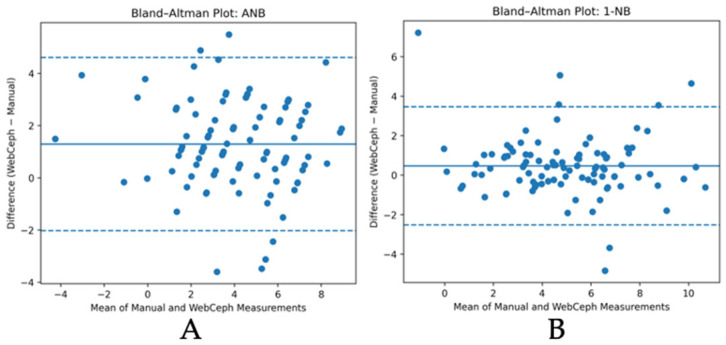
Bland–Altman plots illustrating agreement between manual tracing and automated WebCeph measurements for selected Steiner variables. The central solid line represents the mean difference (bias) between methods, while the upper and lower dashed lines indicate the 95% limits of agreement (mean difference ± 1.96 SD). Each point represents an individual cephalometric radiograph.

Bland–Altman analysis revealed differences between methods and variability across variables (Figure 3).

For skeletal variables, SNA and SNB showed positive mean differences (+3.08° and +3.35°, respectively), with wide limits of agreement. ANB showed a small mean difference (+0.32°) with wide limits of agreement.

Dental measurements showed positive mean differences. For 1–NA and 1–NB, automated values were higher, with limits of agreement shifted above zero in 1–NB.

The interincisal angle showed moderate mean differences with wide limits of agreement. For FMA, the mean difference was small, with wide limits of agreement.

Limits of agreement were calculated as mean difference ± 1.96 × standard deviation (SD) of the differences.

## 4. Discussion

### 4.1. Agreement Versus Statistical Significance

The present study evaluated the agreement between expert manual cephalometric tracing and fully automated analysis using the WebCeph platform, focusing on Steiner measurements. The results demonstrate a clear dissociation between statistical significance and agreement.

Although six of the seven variables showed statistically significant differences, Bland–Altman analysis revealed wide limits of agreement across most parameters, indicating substantial variability at the individual level. This finding confirms that statistical comparisons alone are insufficient to determine whether two methods can be considered interchangeable [24,29].

This discrepancy was particularly evident for ANB. Despite similar mean values, agreement was extremely low (ICC = 0.10), indicating that comparable averages may mask clinically relevant inconsistencies. Similar observations have been reported in previous studies, where AI-based cephalometric systems showed acceptable mean values but limited agreement in individual cases [17,24,29].

The principal contribution of the present study lies not in evaluating the internal functioning of AI algorithms, but in demonstrating that statistically significant and clinically relevant discrepancies may persist between automated and expert manual cephalometric measurements, particularly in selected Steiner variables showing low agreement and wide limits of agreement.

### 4.2. Systematic Bias and Measurement Variability

Bland–Altman analysis revealed both systematic bias and variability between methods.

Skeletal measurements showed consistent overestimation in automated analysis, particularly for SNA and SNB, with mean differences exceeding 3° and wide limits of agreement. Comparable patterns have been described in previous studies, including CBCT-based AI systems, where systematic overestimation of skeletal parameters has been observed [17]. The overestimation observed for SNA and SNB may be explained by small but consistent differences in the localization of cranial base and maxillomandibular landmarks. Both SNA and SNB depend on the spatial relationship between Sella, Nasion, and Point A or Point B, respectively. Therefore, even minor deviations in the identification of Nasion or Sella may simultaneously affect both angles in the same direction. A systematic anterior or superior displacement of Nasion, or subtle differences in Sella localization, could contribute to parallel increases in both SNA and SNB values. Similarly, differences in the identification of Point A and Point B, particularly in cases with anatomical superimposition or reduced cortical definition, may further amplify angular discrepancies.

This pattern suggests that the observed differences may not reflect random measurement error alone, but rather consistent landmark localization behaviour affecting cranial base-related angular measurements. Previous studies comparing AI-assisted cephalometric platforms have also reported variable performance across software systems and cephalometric parameters, indicating that discrepancies may depend on the specific algorithm, training dataset, and landmark definitions used by each platform. Studies comparing CephX, WebCeph, WeDoCeph, and AudaxCeph have shown that AI-based platforms do not perform uniformly across all measurements and that skeletal and dental variables may differ depending on the software evaluated. Therefore, the overestimation observed in the present study should be interpreted as potentially related to WebCeph-specific landmark detection behaviour rather than as a universal tendency of all AI cephalometric systems.

Dental measurements showed the largest discrepancies, particularly for 1–NA and 1–NB, where mean differences exceeded 2 mm. These findings are consistent with previous reports indicating lower reliability of AI-based landmark detection in dental regions, particularly for incisor apices and root-related landmarks [15,22].

The pattern observed for 1–NB, with limits of agreement entirely above zero, indicates a directional bias rather than random variability. From a clinical standpoint, this may be relevant in borderline cases where small differences in incisor position influence treatment decisions.

For FMA, minimal agreement was observed despite a statistically significant mean difference, reflecting the sensitivity of vertical measurements to landmark identification and reference plane construction. This is consistent with the well-established principle that small errors in landmark identification may propagate and amplify in derived angular measurements, particularly when multiple landmarks are involved [30].

### 4.3. Interpretation in the Context of AI-Based Cephalometry

The findings of the present study are consistent with the growing body of evidence indicating that AI-based cephalometric systems show variable performance depending on the type of measurement and anatomical region. Recent systematic evidence has demonstrated that deep learning models can achieve clinically acceptable levels of accuracy for landmark detection under controlled conditions; however, performance remains heterogeneous across studies and anatomical structures [10]. Similarly, previous validation studies have reported that agreement between automated and manual measurements may vary substantially depending on the specific variable analysed [3,31].

From a broader perspective, artificial intelligence is increasingly integrated into medical imaging workflows, improving efficiency, reproducibility, and standardisation, while simultaneously introducing challenges related to validation, interpretability, and clinical oversight [32]. In orthodontics, this transition is particularly relevant given the reliance of diagnosis on precise landmark identification and derived measurements.

From a technical standpoint, most contemporary automated cephalometric systems—including web-based platforms such as WebCeph—are based on deep learning architectures, typically convolutional neural networks trained on large datasets of annotated lateral cephalograms [3,5]. These models detect landmarks by learning hierarchical image features rather than applying predefined anatomical rules, which allows automation but introduces dependency on training data and model generalisation capacity [10].

Fully automated systems have reported mean landmark detection errors typically ranging between approximately 1 and 2 mm, although variability increases depending on dataset heterogeneity and imaging conditions [3,5]. Importantly, these levels of accuracy do not necessarily translate into clinical interchangeability, particularly for angular measurements derived from multiple landmarks [11].

Performance is not uniform across all anatomical regions. Previous studies have consistently shown that landmark detection accuracy is higher for stable skeletal structures, such as cranial base points, and lower for dental and root-related landmarks, where anatomical variability, superimposition, and image ambiguity are greater [10,11]. This pattern is consistent with the present findings, where dental measurements and certain angular variables exhibited lower agreement and greater variability compared with skeletal parameters. This variability likely reflects differences in landmark detectability across anatomical structures. Landmarks associated with incisor apices, mandibular contours, or overlapping craniofacial structures are generally more susceptible to localization error, which may subsequently propagate into derived Steiner measurements.

The behaviour of automated systems should also be interpreted in light of their operational design. As indicated in software documentation, these platforms perform fully automated landmark detection but explicitly acknowledge that predicted landmark positions may deviate from their true anatomical location, requiring user verification prior to clinical use. This reinforces the concept that such systems are not intended to function as fully autonomous diagnostic tools, but rather as assistive technologies that complement clinician judgement [32].

Furthermore, automated landmark detection is influenced by factors such as image quality, patient positioning, and case complexity, which may further contribute to variability under real-world clinical conditions [10,11]. These factors are particularly relevant in routine clinical environments, where imaging conditions are less controlled than in model training datasets.

Previous literature has suggested that the relative opacity of certain AI-based cephalometric systems may limit clinician interpretation of landmark localization discrepancies, particularly when disagreement between automated and manual measurements arises [32]. As a result, reliance on automated outputs without critical evaluation may introduce diagnostic uncertainty.

Taken together, these considerations help explain the systematic differences and variability observed in the present study. The tendency of automated measurements to produce higher values, particularly in dental variables, may reflect consistent landmark displacement rather than random error, a phenomenon that has been previously described in AI-based cephalometric analyses [11]. The present findings support cautious interpretation of AI-generated cephalometric measurements, particularly in variables showing low agreement or wide limits of agreement.

### 4.4. Clinical Implications

Automated cephalometric analysis offers clear advantages in terms of efficiency, accessibility, and integration into digital workflows. These systems can substantially reduce analysis time and may improve consistency by minimizing operator-dependent variability, particularly in high-throughput or standardized clinical environments [10,32].

However, the present findings indicate that these advantages do not necessarily translate into clinical interchangeability with expert manual tracing. Despite acceptable mean values in some parameters, the observed variability and limited agreement at the individual level restrict the use of automated outputs as standalone diagnostic tools. This is particularly relevant for variables directly influencing treatment decisions, such as incisor position and sagittal skeletal relationships, where small discrepancies may alter clinical interpretation [11,33].

From a clinical perspective, automated systems should therefore be considered assistive tools rather than replacements for clinician judgement. Their role may be particularly appropriate in screening, educational contexts, and preliminary assessment, where rapid estimation of cephalometric parameters is beneficial. However, in definitive diagnosis and treatment planning—especially in borderline or complex cases—manual verification of landmark identification and derived measurements remains essential [11,33].

These findings align with the broader paradigm of AI implementation in healthcare, where automated systems are designed to augment clinical decision-making rather than replace it, requiring appropriate validation, supervision, and contextual interpretation [32].

Accordingly, clinicians should adopt a critical approach when interpreting AI-generated cephalometric outputs, recognizing both their potential and their limitations. Future integration of these systems into clinical workflows will likely depend on improvements in model robustness, transparency, and performance across diverse anatomical conditions.

### 4.5. Limitations and Future Directions

This study has several limitations. First, the analysis was restricted to Steiner variables and a single AI platform, which may limit generalizability to other analytical systems or software. An additional limitation of the present study is the absence of formal quantitative intraobserver and interobserver reliability analysis for manual tracing. Although all measurements were performed under standardized conditions and reviewed by experienced orthodontists, future studies should incorporate repeated-measures reproducibility assessment to further strengthen the manual reference standard. Second, the sample was not stratified according to skeletal pattern or malocclusion type, factors that may influence algorithm performance.

Future studies should investigate AI performance across different skeletal classes and clinical scenarios, as well as evaluate hybrid approaches combining automated landmark detection with clinician validation.

In addition, further research should explore the influence of image acquisition parameters and radiographic quality on AI performance, as well as assess longitudinal consistency in clinical monitoring contexts [18]. The present study evaluated discrepancies at the measurement level rather than at the individual landmark level. Consequently, it was not possible to determine which specific anatomical landmarks contributed most substantially to the observed differences between manual and automated analyses. Since cephalometric measurements are directly dependent on accurate landmark localization, small deviations in landmarks such as Sella, Nasion, Point A, or incisor apices may propagate into clinically relevant angular and linear discrepancies. Future studies should therefore incorporate landmark-level error mapping and spatial accuracy analysis to better characterize the origin of AI-related measurement variability.

Although radiographs with inadequate quality or positioning errors were excluded to ensure methodological standardization, this may limit the extrapolation of the findings to routine clinical settings, where image quality and anatomical variability are often less controlled. Since previous evidence suggests that AI performance may deteriorate in low-quality or non-standardized radiographs, future studies should specifically evaluate the robustness of automated cephalometric systems under real-world imaging conditions.

## 5. Conclusions

Fully automated cephalometric analysis using the WebCeph platform demonstrated statistically significant differences compared with expert manual tracing across most evaluated Steiner variables. Although some parameters showed relatively small mean differences, agreement at the individual level was inconsistent and ranged from low to moderate across variables.

Automated measurements showed a systematic tendency toward higher values, particularly in selected skeletal and dental parameters, with wide limits of agreement observed for several measurements. These findings suggest that automated and manual measurements cannot be considered fully clinically interchangeable within the conditions evaluated in the present study.

While WebCeph may provide useful support for workflow efficiency, preliminary assessment, and educational applications, careful professional verification of AI-generated measurements remains advisable, particularly in clinically relevant variables showing limited agreement or potential diagnostic impact.

Further studies comparing multiple AI platforms, evaluating landmark-level discrepancies, and assessing performance across different skeletal and clinical conditions are warranted.

## Figures and Tables

**Table 1:** Comparison of cephalometric measurements obtained by manual tracing and automated analysis (WebCeph).

Variable	Manual (Mean ± SD)	Automated (Mean ± SD)	Mean Difference (Auto − Manual)	*p*-Value	ICC Agreement
SNA (°)	79.03 ± 3.92	82.11 ± 4.61	+3.08	0.00018	0.55
SNB (°)	73.03 ± 3.22	76.38 ± 3.89	+3.35	<0.001	0.60
ANB (°)	5.47 ± 2.91	5.78 ± 3.01	+0.32	0.56	0.10
1–NA (mm)	4.70 ± 2.12	6.35 ± 2.44	+1.65	<0.00001	0.40
1–NB (mm)	4.67 ± 1.98	6.96 ± 2.31	+2.29	<0.00001	0.32
1/1 (°)	128.87 ± 5.34	131.84 ± 6.02	+2.97	<0.00001	0.52
FMA (°)	25.93 ± 3.11	26.87 ± 3.54	+0.94	0.01222	0.01

**Table 2:** Bland–Altman agreement parameters between manual tracing and automated WebCeph cephalometric measurements.

Variable	Mean Difference (Auto − Manual)	SD of Differences	Lower Limit of Agreement	Upper Limit of Agreement
SNA (°)	+3.08	3.92	−4.60	+10.76
SNB (°)	+3.35	3.22	−2.96	+9.66
ANB (°)	+0.32	2.91	−5.38	+6.02
1–NA (mm)	+1.65	0.92	−0.15	+3.45
1–NB (mm)	+2.29	0.96	+0.41	+4.17
1/1 (°)	+2.97	2.65	−2.22	+8.16
FMA (°)	+0.94	1.92	−2.82	+4.70

## Data Availability

The data supporting the findings of this study are available from the corresponding author upon reasonable request. The data are not publicly available due to privacy and institutional restrictions.
